# Correlation of High-Risk Soft Tissue Sarcoma Biomarker Expression Patterns with Outcome following Neoadjuvant Chemoradiation

**DOI:** 10.1155/2018/8310950

**Published:** 2018-02-28

**Authors:** John M. Kane, Anthony Magliocco, Qiang Zhang, Dian Wang, Alex Klimowicz, Jonathan Harris, Jeff Simko, Thomas DeLaney, William Kraybill, David G. Kirsch

**Affiliations:** ^1^Roswell Park Cancer Institute, Buffalo, NY, USA; ^2^H. Lee Moffitt Cancer Center, Tampa, FL, USA; ^3^NRG Oncology Statistics and Data Management Center, Philadelphia, PA, USA; ^4^Rush University Medical Center, Chicago, IL, USA; ^5^Tom Baker Cancer Centre, Calgary, AB, Canada; ^6^University of California, San Francisco, CA, USA; ^7^Massachusetts General Hospital, Boston, MA, USA; ^8^The Ohio State University, Columbus, OH, USA; ^9^Duke University Medical Center, Durham, NC, USA

## Abstract

**Background:**

Sarcoma mortality remains high despite adjuvant chemotherapy. Biomarker predictors of treatment response and outcome could improve treatment selection.

**Methods:**

Tissue microarrays (TMAs) were created using pre- and posttreatment tumor from two prospective trials (MGH pilot and RTOG 9514) of neoadjuvant/adjuvant MAID chemotherapy and preoperative radiation. Biomarkers were measured using automated computerized imaging (AQUA or ACIS). Expression was correlated with disease-free survival (DFS), distant disease-free survival (DDFS), and overall survival (OS).

**Results:**

Specimens from 60 patients included 23 pretreatment (PRE), 40 posttreatment (POST), and 12 matched pairs (MPs). In the MP set, CAIX, GLUT1, and PARP1 expression significantly decreased following neoadjuvant therapy, but p53 nuclear/cytoplasmic (N/C) ratio increased. In the PRE set, no biomarker expression was associated with DFS, DDFS, or OS. In the POST set, increased p53 N/C ratio was associated with a significantly decreased DFS and DDFS (HR 4.13, *p*=0.017; HR 4.16, *p*=0.016), while increased ERCC1 and XPF expression were associated with an improved DFS and DDFS. No POST biomarkers were associated with OS.

**Conclusions:**

PRE biomarker expression did not predict survival outcomes. Expression pattern changes after neoadjuvant chemoradiation supports the concepts of tumor reoxygenation, altered HIF-1*α* signaling, and a p53 nuclear accumulation DNA damage response.

**Clinical Trial Registration:**

NRG Oncology RTOG 9514 is registered with ClinicalTrials.gov. The ClinicalTrials.gov Identifier is NCT00002791.

## 1. Introduction

Despite major improvements in local control/limb salvage, survival for “high-risk” soft tissue sarcomas (STSs) has not significantly changed over time. Almost half of patients with a large, deep, high-grade sarcoma (stage III) will die within 5 years of their diagnosis. Consequently, the potential benefits of adjuvant chemotherapy have been explored. Several studies have reported improvements in overall survival of 4–19% [1–3]. However, toxicity has been significant, including both acute hematologic toxicity and late myelodysplasia/leukemia [2, 4, 5]. In addition, it has been impossible to identify which subset of “high-risk” patients truly benefit from adjuvant chemotherapy based upon standard prognostic variables (age, location, histologic subtype, size, and grade) [1].

Beginning in 1989, Massachusetts General Hospital (MGH) performed a pilot trial of neoadjuvant MAID chemotherapy (mesna, Adriamycin, ifosfamide, and dacarbazine), 44 Gy interdigitated preoperative radiation, and adjuvant systemic chemotherapy in 48 patients with high-risk extremity STSs (high grade, ≥8 cm) [4]. Actuarial 5-year overall survival (OS) was 87%, significantly higher than that of a corresponding historical control group. Long-term follow-up for these patients was also available as part of a larger study [6]. At a median follow-up of 46 months, 5-year OS was still 86%. Based upon the promising results of the MGH pilot trial, the Radiation Therapy Oncology Group (RTOG) opened trial 9514 in 1997, a phase II study of neoadjuvant chemotherapy and radiation for “high-risk” soft tissue sarcomas of the extremities and trunk [5]. Sixty-four patients were treated using a regimen almost identical to that in the MGH pilot trial. With long-term follow-up, the estimated 5-year OS was an impressive 71.2% [7].

As part of NRG Oncology RTOG 9514, the original biopsy and surgical resection specimens for many of the participating patients were stored in the RTOG Tissue Bank. In addition, one of the principal investigators for the MGH pilot trial was also involved in NRG Oncology RTOG 9514, providing access to many of the pathology specimens from the MGH pilot trial. Both studies had long-term follow-up information on a group of large, high-grade STS patients treated with the same neoadjuvant chemoradiation/adjuvant chemotherapy regimens. This afforded a unique opportunity to construct tissue microarrays (TMAs) of the tumor specimens, which could be used to potentially correlate candidate biomarker expression with outcome in a uniform cohort of “high-risk” STS patients.

## 2. Methods

### 2.1. Specimen Acquisition

After obtaining institutional review board approval from MGH, available diagnostic biopsy and surgical resection specimens from the pilot trial were pathologically reviewed to assure the presence of viable tumor. The representative paraffin blocks were then sent to the RTOG Tissue Bank at the University of San Francisco. Representative 0.6 mm punch biopsies were obtained in order to create a TMA, and the blocks were returned to the parent institution. Many of the specimens from NRG Oncology RTOG 9514 were already housed in the RTOG Tissue Bank as part of the original protocol. An attempt was made to obtain any missing specimens by contacting the treating institutions.

The original pretreatment diagnostic biopsy for patients in both trials was typically a core needle or limited incisional biopsy specimen. Therefore, it was only a representative portion of a larger tumor. In contrast, the final surgical specimen following neoadjuvant therapy often contained tissue blocks from several different areas. To minimize the sampling error from biomarker expression heterogeneity and to maximize the acquisition of adequate quality tumor for immunohistochemistry (IHC) analysis (e.g., nonnecrotic tissue), the parent institution was asked to provide two blocks representing different areas of assessable tumor (ideally, one central and one peripheral). H&E sections from these blocks were reviewed, and areas of highest tumor concentration circled to act as guides for punching the TMA cores. As the potential for tumor heterogeneity was considered, TMAs were constructed in triplicate.

### 2.2. Candidate Biomarkers

All of the patients in this study came from either the MGH pilot trial or RTOG 9514, which were large, deep, “high-risk” STSs. Therefore, some of the biomarkers chosen were previously associated with STS outcome in smaller, more heterogeneous studies. In addition, all of the patients had received neoadjuvant chemoradiation. Consequently, other biomarkers had potential relationships to the cellular responses to chemoradiation damage. Although many biomarkers were available for assessment, nine candidate biomarkers were chosen for this initial TMA analysis for the reasons outlined above.

*Ki67* is a nuclear nonhistone protein that is expressed in proliferating cells [8]. Increased Ki67 expression in STSs has been associated with a decreased metastasis-free survival [9, 10].

*p53* is a multifunctional “tumor suppressor” protein, which can be induced by DNA damage to cause cell cycle arrest to allow for DNA damage repair, activate DNA repair proteins, and initiate apoptosis [11]. p53 mutations and overexpression on IHC, especially as assessed by N-terminal binding antibodies, are associated with decreased STS survival [12, 13].

*Ataxia-telangiectasia mutated (ATM)* kinase is a serine-threonine kinase that mediates cell cycle checkpoint control following exposure to agents that produce double-stranded DNA breaks, such as ionizing radiation [14]. ATM-dependent arrest of the cell cycle in G1 following radiation is through activation of p53 [15].

*Poly (ADP-ribose) polymerase-1 (PARP1)* is activated by DNA damage, playing a role in DNA base excision repair, but can also regulate transcription [16]. Loss of PARP1 activity leads to enhanced cancer cell death. Doxorubicin has been anecdotally shown to decrease PARP1 expression/activity [17].

*Excision repair cross-complementation group 1 (ERCC1)* is a rate-limiting protein in the nucleotide excision repair and interstrand crosslink repair pathways, including removing platinum chemotherapy adducts [18]. In STS patients undergoing trabectedin therapy, high expression correlated with improved progression-free survival and OS [19].

*Xeroderma pigmentosum group F-complementing protein (XPF)* is the catalytic component of the ERCC1 structure-specific DNA repair endonuclease complex [18].

*Carbonic anhydrase IX (CAIX)* is a transmembrane protein that catalyzes the hydration of carbon dioxide to carbonic acid, modulating pH [20]. Significant upregulation can occur with tumor hypoxia [21]. Expression has been associated with a decreased disease-specific survival and OS in large, deep, high-grade STSs [22].

*Glucose transporter 1 (GLUT1)* facilitates the transport of glucose across the plasma membranes of mammalian cells. It can be upregulated by hypoxic conditions, facilitating tumor cell generation of ATP via anaerobic glycolysis [23]. Bone and STS patients with glut-1 overexpression had a significantly worse OS as compared to those without overexpression [24].

*Hypoxia-inducible factor 1-alpha (HIF-1α)* is a subunit of the transcription factor that regulates the cellular responses to hypoxia and may increase expression of proteins necessary for the development of metastases [25–27]. Increased HIF-1*α* expression in STSs has been associated with a shorter OS [28]. HIF-1*α* can also mediate resistance to radiation therapy [29].

### 2.3. TMA Construction, Staining, and Scoring

TMAs were constructed, stained, and scored using commercially available reagents and previously described, well-validated techniques [30–33].

### 2.4. TMA Construction

Two consecutive representative sections were recut from each specimen block. One was banked for future reference, if necessary. The other recut slide was stained with hematoxylin and eosin and used to determine the optimal locations for the 0.6 mm core biopsies of the specimen block. For the smaller-sized pretreatment biopsy specimens, one block was processed in this manner. For the larger posttreatment surgical resection specimens, if available, two blocks from noncontiguous areas of the tumor were processed. This algorithm produced 2-3 cores/patient (1 pretreatment and 1-2 posttreatment) on the TMA block. The blocks were arrayed using consecutive patients in order. Representative tissues with known IHC positivity to each of the study biomarkers were chosen by the Tissue Bank and also placed on the master TMA block to serve as positive controls.

As previously noted, three copies of the TMA were made by taking punches from different parts of the available tumor and stained with each marker to allow for consideration of expression heterogeneity across different areas of the tumor.

Construction of the TMA block was performed using the automated instruments at the RTOG Tissue Bank (Beecher Instruments, Sun Prairie, WI). Recipient paraffin blocks were made using standard jumbo metal molds commonly used for tissue embedding with standard plastic histology cassettes on top (Sakura Finetek, Torrance, CA). Type 9 embedding paraffin (Richard-Allan Scientific, Kalamazoo, MI) was used to make the paraffin recipient TMA block. A supplied H&E slide marked to represent the area of interest and its corresponding paraffin tissue block was used to accurately punch out the donor cores for each case and placed into the TMA. Heat strips were glued to the recipient block holder on the TMA instrument. The recipient block was then heated to approx. 100°F.

After each array block was constructed, a glass slide heated up to 80°C was set on the warmed block and allowed to melt the surface. The heated glass slide set the punches, eliminating the oven step used in the original method for array construction. The glass slide and array block were then turned over and allowed to cool on a room temperature tabletop for 5 minutes before chilling on an ice tray (slide down) before separating. The microtome used was the Microm HM 355-S (Thermo Fischer Scientific, Waltham, MA). The microtome chuck was adjusted manually to the flat surface of the array block. Since the block warmer was used when punching the array blocks, the punch surface was already flat, eliminating the need to trim into the block. Sections were taken almost directly off the surface. TMA blocks were sectioned at 4 microns and gently placed on a water bath at 38°C. Individual sections were maneuvered onto plus-charged slides with a probe. For quality control, a section from each TMA was stained with H&E. Each TMA core was checked to verify the presence or absence of tumor by the biobank pathologist (JPS), followed by a subsequent recheck by the pathologist performing the immunohistochemical studies (TM).

### 2.5. Fluorescence Immunohistochemistry

TMA sections (4 microns) were deparaffinized in xylene, rinsed in ethanol, and rehydrated as previously described [32]. Heat-induced epitope retrieval (HIER) was performed using a Decloaking Chamber (Biocare Medical, Concord, CA) for all target biomarkers by heating slides to 121°C for either 3 or 6 minutes, in either a citrate-based (pH 6.0) target retrieval solution (S1699, DAKO, Mississauga, Canada), or a Tris/EDTA-based (pH 9.0) target retrieval solution (S2367, DAKO).  Supplementary 2 summarizes all target antibody specifics and their HIER conditions.

Slides were then processed using a DAKO Autostainer. Endogenous peroxidase activity was quenched with a 10-minute incubation of peroxidase block (K4007, DAKO), followed by a 15-minute protein block (SignalStain®; 8112L, Cell Signaling, Danvers, MA) to prevent nonspecific antibody binding.

All primary antibodies were diluted in SignalStain and applied for 60 minutes at room temperature along with either rat anti-vimentin (MAB2105, clone 280618, 1:100, R&D Systems, Minneapolis, MN, USA) or rabbit anti-vimentin (2707-1, clone EPR3776, 1:250, Epitomics, Burlingame, CA) to identify tumor cells. Slides were washed in Tris-buffered saline and Tween® 20 (TBST) wash buffer (S3006, DAKO) and then treated for 60 minutes with either goat anti-rabbit EnVision + (K4011, DAKO) or goat anti-mouse EnVision + (K4007, DAKO) secondary antibody. Either Alexa-488-conjugated goat anti-rat antibody (A-11006, polyclonal, 1:200, Invitrogen, Burlington, ON, Canada) or Alexa-555-conjugated goat anti-rabbit antibody (A-21429, polyclonal, 1:200, Invitrogen) was applied along with the anti-rabbit and anti-mouse secondary antibodies, respectively, to detect vimentin. Slides were then treated for 5 minutes with a TSA-Plus Cy5 tyramide signal amplification reagent (NEL745B001KT, PerkinElmer, Waltham, MA), coverslipped using ProLong Gold antifade mounting medium with 4′,6-diamidino-2-phenylindole (DAPI) (P36935, Invitrogen), and stored at 4°C.

### 2.6. Automated Image Acquisition and Analysis

Compartment-specific expression of all biomarkers was quantified using the HistoRx AQUA® platform (Branford, CT). Automated image acquisition was performed using the HistoRx PM-2000™ slide scanner, and digital images were analyzed using AQUAnalysis® software version 2.3.4.1 as previously described [33]. Briefly, seamless high-resolution images were acquired using an 8-bit monochrome TDI line-image capture camera with filters specific for DAPI to define the nuclear compartment, either fluoroscein isothiocyanate (FITC) or Cyanine 3 (Cy3) to define the vimentin-positive tumor cytosolic compartment, and Cy5 to define all target markers. A tumor-specific mask was generated to distinguish cancer cells from surrounding stromal tissue by thresholding the vimentin images to create a binary mask that identified the presence or absence of tumor cells by the presence of a pixel that was “on” or “off,” respectively.

Images were cropped to exclude unusable areas from final analysis and then processed using optimized threshold values. Images were validated according to the following: (1) >10% of the tissue area is vimentin positive and (2) >50% of the image was usable (i.e., not compromised due to overlapping or out of focus tissue).

Compartment-specific AQUA scores, representing protein expression for all markers, were calculated as the average concentration of Cy5 pixel intensity within the compartment area for each TMA core. For each patient sample, the average compartment-specific AQUA score over triplicate cores was used to define the tumor score (tAQUA). Representative staining for CAIX and GLUT1 is shown in Supplementary 1.

### 2.7. 3′-Diaminobenzidine Tetrahydrochloride (DAB) Immunohistochemistry (Ki67 Only)

Deparaffinized and rehydrated TMA sections underwent HIER using the PT-Link (DAKO) by heating slides at 97°C for 20 minutes in DAKO EnVision™ FLEX, Low pH (Link) target retrieval solution. After cooling to 65°C, slides were then placed in 1x EnVision FLEX wash buffer for 5 minutes prior to running the slides on the DAKO Autostainer Link 48. The FLEX monoclonal mouse anti-human Ki67 antigen (clone MIB-1) ready-to-use antibody (DAKO, IR626) for the Link platform was used following the manufacturer's specifications using EnVision FLEX reagents (DAKO, K8002) with counterstaining performed on the autostainer using hematoxylin (Link) (DAKO, K8008).

All images were reviewed by one of the study pathologists (TM) as a quality control step in data acquisition. Immunohistochemical staining was quantitatively assessed using ACIS® III Automated Cellular Imaging System (DAKO). Briefly, the ACIS III digitizes and reports a region score using proprietary software to generate a specific algorithm for Ki67 that identifies color thresholds (blue (hematoxylin overlay) and light brown and dark brown (Ki67DAB)) in manually selected regions containing tumor. The percent positive ACIS III score was then calculated by taking the total brown staining area and dividing it by the combined total blue and brown staining areas.

### 2.8. Outcome Data and Statistical Analysis

The demographic and updated outcome data for all of the patients that participated in NRG Oncology RTOG 9514 were already contained within the RTOG clinical trial database. Corresponding data for the patients in the MGH pilot trial were obtained from the MGH database, reformatted, and merged with the NRG Oncology RTOG 9514 data. Patient demographic information included age, gender, STS histology, tumor size, anatomic location, the pathologic response to neoadjuvant therapy as per the resection specimen, local recurrence, distant recurrence, and vital status at the time of the last follow-up.

For the purpose of statistical analysis, there were 3 cohorts of TMA specimens. The “pretreatment” (PRE) group consisted of specimens obtained at the time of STS diagnosis/prior to starting preoperative chemoradiation. The “posttreatment” (POST) group was specimens from the definitive surgical resection (following neoadjuvant chemoradiation). Based upon the presence of both pretreatment biopsy and surgical resection specimens for an individual patient, there was also a cohort of “matched pairs” (MPs). The patients with a pathologic complete response (PCR) were excluded from the POST and MP group analyses as there was no viable tumor for the POST TMA construction. Markers were analyzed as log-transformed continuous variables.

Assessed outcome measures included disease-free survival (DFS), distant disease-free survival (DDFS), and overall survival (OS). Failure for DFS was defined as local, regional, or distant relapse, or death due to any cause. Failure for DDFS was defined as distant relapse or death due to any cause. Failure for OS was defined as death due to any cause. DFS, DDFS, and OS were measured from the date of surgery to the date of failure or last follow-up for censored patients. The patients that progressed or died prior to surgery were excluded from analysis. Rates for DFS, DDFS, and OS were estimated by the Kaplan-Meier method. Hazard ratios were estimated by Cox models. Change of marker levels from pre- to posttreatment was compared using the nonparametric Wilcoxon signed-rank test.

## 3. Results

### 3.1. Patient Demographics and Outcome

Specimens for TMA construction were obtained for 61 patients from the combined MGH pilot trial/NRG Oncology RTOG 9514 participants. Following pathologic review, specimens from 60 patients were deemed adequate for TMA construction (37 from NRG Oncology RTOG 9514 and 23 from the MGH). Fifty-three patients had at least one marker value available. Two patients were excluded from analysis (1 not meeting inclusion/exclusion criteria; 1 disease progression prior to surgery), leaving 51 analyzable patients. In terms of the cohorts for analysis, there were 23 PRE patients, 40 POST patients, and 12 MPs.

**Figure 1:**
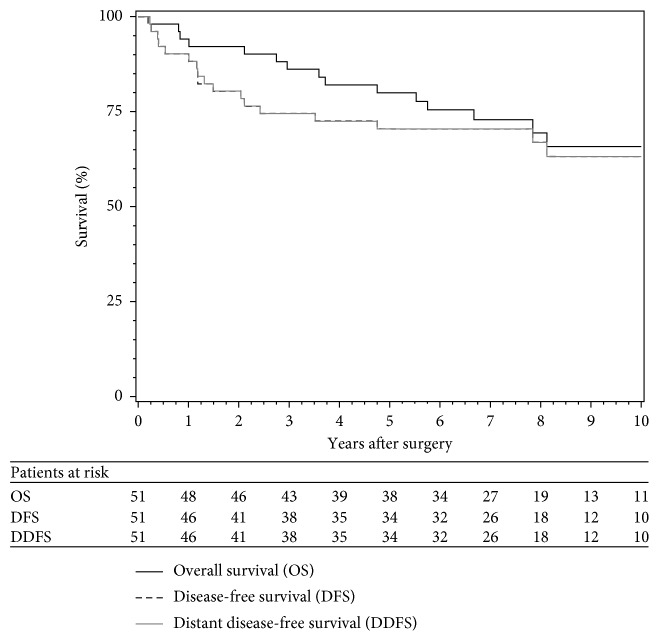
Kaplan-Meier estimates for disease-free survival, distant disease-free survival, and overall survival for deep, high-grade soft tissue sarcoma patients (with at least one biomarker value) treated with neoadjuvant chemoradiation.

The clinical and pathologic data for all patients are summarized in Supplementary 3. The median age for the patients represented on the TMA was 48 years (range 21–77), and 56.9% were male. Median tumor size was 14 cm (range 8.2–35), and 76.5% were located on the lower extremity/buttock. The most common STS subtypes were 45.1% undifferentiated pleomorphic sarcoma (malignant fibrous histiocytoma), 13.7% non-well-differentiated liposarcoma, 11.7% leiomyosarcoma, 5.9% malignant peripheral nerve sheath tumor, and 3.9% synovial sarcoma. Negative margin wide resection was achieved in 90.2%. Eighty-two percent of patients received all 3 cycles of preoperative MAID chemotherapy and 65% received 3 postoperative cycles. The complete follow-up and outcome data are shown in Supplementary 4. The median follow-up for surviving patients was 7.8 years (range 1.8–17.6). The 5-year estimates for DFS, DDFS, and OS were 70.4% (95% CI 57.9–83.0), 70.4% (57.9–83.0), and 79.9% (68.8–91.1), respectively (Figure 1).

### 3.2. Changes in Biomarker Expression following Neoadjuvant Chemoradiation

The changes in tumor biomarker expression following neoadjuvant chemoradiation in the 12 MP patients are listed in Table 1. There were statistically significant decreases in the expression of nuclear, cytoplasmic, and tumor mask CAIX (*p*=0.023, 0.039, and  0.016, resp.), tumor mask GLUT1 (*p*=0.047), and nuclear PARP1 (*p*=0.031). p53 nuclear/cytoplasmic ratio also significantly increased (*p*=0.047) following preoperative therapy. There were no significant changes in Ki67, ATM, ERCC1, XPF, or HIF-1*α*.

### 3.3. Biomarker Expression and Survival

The PRE group biomarker expression data are listed in Supplementary 5. There was no predictive association between PRE group biomarker expression and DFS, DDFS, or OS (data not shown). The POST group biomarker expression data are listed in Supplementary 6. For POST group biomarker expression, increased p53 nuclear/cytoplasmic (N/C) ratio was associated with a significantly decreased DFS (HR 4.13 (95% CI: 1.29–13.17), *p*=0.017). Increased ERCC1 tumor mask and XPF nuclear expression were associated with an improved DFS (HR 0.30 (95% CI: 0.09–0.97), *p*=0.044; HR 0.01 (95% CI: 0.00–0.92), *p*=0.046, resp.). The entire POST group DFS analysis is shown in Table 2. No other POST group biomarkers were associated with DFS. Similar to DFS, increased p53 nuclear/cytoplasmic (N/C) ratio (HR 4.16 (95% CI: 1.31–13.23), *p*=0.016) and a low expression of ERCC1 (HR 0.30 (95% CI: 0.09–0.98), *p*=0.046) and XPF (HR 0.01 (95% CI: 0.00–0.93), *p*=0.046) were associated with a decreased DDFS (Table 3). There was no relationship between POST biomarker expression and OS (data not shown).

## 4. Discussion

In light of the associated toxicity of cytotoxic chemotherapy, better predictors of poor survival in high-risk STS patients could potentially justify systemic treatment-related morbidity in a subset of this group. The tumor specimens from the MGH pilot trial and NRG Oncology RTOG 9514 provided a unique opportunity to create TMAs from a uniform cohort of high-risk STS patients with complete prospective follow-up data. The patients were also treated with almost identical neoadjuvant chemoradiation regimens. Unfortunately, there were no pretreatment expression patterns in our chosen biomarkers that correlated with DFS, DDFS, or OS. However, there were a few posttreatment biomarkers associated with the risk for distant recurrence: p53 N/C ratio, ERCC1, and XPF. If these biomarkers were associated with the risk for distant recurrence after neoadjuvant radiation therapy alone, then they could be tested to stratify patients for postoperative chemotherapy. There were also some intrapatient changes in tumor biomarker expression following neoadjuvant therapy that may better elucidate the biologic mechanisms underlying STS responses to chemoradiation.

In resected high-grade STSs without neoadjuvant therapy, Maseide et al. observed that the expression of CAIX correlated with decreased disease-specific survival and OS [22]. In our study, neither PRE nor POST CAIX expression was associated with outcome. Interestingly, we did observe that preoperative chemoradiation led to a significant decrease in both CAIX and GLUT1 tumor expression in the MP analysis. These findings would fit with a chemoradiation-related tumor reoxygenation phenomenon [34]. Portions of tumors (especially STS) can be hypoxic, making them less responsive to radiation. As tumor cells die in response to therapy, other hypoxic cells within the tumor will obtain more oxygen. Expression of CAIX and GLUT1 can be induced by HIF-1*α* [35, 36]. A decrease in HIF-1*α* secondary to tumor reoxygenation resulting from neoadjuvant therapy could produce the observed decreases in CAIX and GLUT1. However, we did not observe a decrease in HIF-1*α* in our MP group. Therefore, it is possible that the decreased expression of CAIX and GLUT1 after neoadjuvant therapy reflects altered HIF-1*α* signaling in response to chemoradiation, such as doxorubicin, rather than expression changes only attributable to hypoxia [37, 38].

Given the roles of PARP1, ERCC1, and XPF in the repair of DNA damage, one might expect that increased expression would enhance tumor survival. In our study, increased primary tumor POST expression of both ERCC1 and XPF was associated with an increased DFS and DDFS. This finding is somewhat similar to ERCC1 results by Rodrigo et al. in 78 high-grade “locally advanced” soft tissue sarcoma patients who received 4 cycles of neoadjuvant doxorubicin-cisplatin-ifosfamide chemotherapy [39]. Although there was no significant correlation of ERCC1 negative versus positive tumors with DFS in that study (median DFS 3.2 versus 7 years, *p* ≤ 0.19), median OS for ERCC1-positive tumor was not reached as compared to 6.6 years for negative tumors (*p* ≤ 0.058). We observed a decrease in PARP1 expression after neoadjuvant therapy in the MP group, but there was no correlation between PRE or POST PARP1 expression and outcome. Increased PARP1 expression has been associated with decreased DFS and DDFS in other cancers, such as serous ovarian carcinoma and breast cancer [40, 41]. As previously noted, doxorubicin therapy has been associated with decreases in PARP1, which may explain our PARP1 findings [17].

Another interesting finding was the increase in p53 N/C ratio following neoadjuvant therapy in the MP group combined with an increased POST p53 N/C ratio correlating with a decreased DFS and DDFS. These results raise the possibility that chemoradiation may have increased the expression of preexisting mutant p53. Mutant p53, with loss of function, can have a positive feedback loop with respect to p53 expression, resulting in increased nuclear accumulation [42]. It also has a higher N/C ratio as there is less cytoplasmic background due to saturation of the camera from the strong nuclear expression. Use of the N/C ratio helps to normalize differences on a case-by-case basis, and it also relates to changes in localization of the protein. The fact that the p53 N/C ratio findings for DFS and DDFS were statistically significant supports a hypothesis that sarcomas with mutant p53 may be at increased risk of metastasis. Alternatively, it is conceivable that the increased accumulation of p53 following neoadjuvant therapy reflects activation of wild-type p53 that accumulates in the nucleus following DNA damage. In this scenario, the presence of wild-type p53 would correlate with worse outcome after treatment, which has been reported for preclinical mouse models of breast cancer treated with doxorubicin [43]. Future studies that include p53 gene sequencing and evaluation of the p53 N/C ratio after neoadjuvant therapy will be needed to differentiate between these two possibilities.

There are several limitations to our study. The first is that, although this is a very homogeneous, uniformly treated subset of “high-risk” STSs, the total number of patients is very small. This would potentially diminish the ability to identify any statistically significant associations between biomarker expression and outcome. As with many pathologic specimen-based analyses, we were only able to acquire adequate specimens to construct the TMA from 59 out of 112 potential patients from the combined MGH/NRG Oncology RTOG 9514 trials. Many institutions were unwilling to release the archival diagnostic pathologic material. The original pretreatment biopsy specimens were frequently very limited in size, often just a core needle biopsy, and some were performed at institutions not participating in either trial (prior to the referral of the patient for trial enrollment). When limited samples from a large tumor are used to construct a TMA, it is also possible that tumor heterogeneity will not be adequately represented. This is especially true for the rather diminutive pretreatment core needle biopsy specimens. Alternatively, one could contend that using a single small core needle biopsy specimen from a newly diagnosed sarcoma would be more representative of what would happen in a “real-world” clinical practice where only a very small, somewhat random biopsy sample from the tumor would be available to perform biomarker testing for pretreatment prognostication. If there was a complete pathologic response to neoadjuvant therapy (which was 27% in NRG Oncology RTOG 9514), it also meant that there was no POST tumor specimen to analyze [5]. Although we “lumped together” the different STS subtypes due to the small number of available cases, there are likely inherent differences in biomarker expression amongst the various histologies. The small number of matched pair specimens also limited the ability to identify intrapatient changes in tumor biomarker expression following neoadjuvant therapy. Finally, although there was a fairly long median follow-up, the overall small sample size combined with very good patient survival means that there were relatively few adverse outcome events, which limited the statistical power of our study.

In conclusion, the PRE expression of none of our candidate pretreatment biomarkers was not associated with survival in this cohort of STS patients treated with neoadjuvant chemoradiation. Therefore, we remain unable to identify a subset of truly high-risk STS patients at the time of diagnosis who would be optimal candidates for neoadjuvant chemoradiation prior to surgical resection. Some tumor biomarker expression pattern changes after neoadjuvant chemoradiation do support the concepts of tumor reoxygenation or altered HIF-1*α* signaling. In addition, we observed nuclear accumulation of p53 after neoadjuvant therapy, which may reflect a response of p53 to DNA damage. Hopefully, our clinically well-annotated, high-risk STS TMAs will be an extremely useful collaborative resource for future candidate biomarker analyses, either for prognostication or to assess the suitability for novel targeted therapies.

## Figures and Tables

**Table 1:** Changes in tumor biomarker expression following neoadjuvant chemoradiation in 12 “matched pair” large, deep, high-risk soft tissue sarcoma patients.

Marker	Pretreatment value		Posttreatment value		*p* value
	Mean	SD	Mean	SD	
ACIS Ki67					
Percentage	15.91	19.78	7.65	7.30	0.0674
p53					
Nuclear/cytoplasmic ratio	1.44	0.22	1.90	0.74	0.0469
ATM					
Nuclear	8349.36	1920.35	7870.75	2114.36	0.4961
Cytoplasm	3811.69	2121.29	3116.21	1035.73	0.4258
Tumor mask	5289.39	2400.71	4412.80	1205.37	0.3594
PARP1					
Nuclear	7042.80	1361.76	5533.59	1198.18	0.0313
Cytoplasm	3281.97	798.55	2293.63	471.64	0.0938
Tumor mask	4354.82	1092.11	3263.65	674.80	0.0625
ERCC1					
Nuclear	9433.46	2247.20	8586.98	1658.74	0.3594
Cytoplasm	3046.29	1563.80	2197.19	917.54	0.0977
Tumor mask	5150.35	1600.28	4206.67	1335.08	0.0977
XPF					
Nuclear	8772.27	1076.08	8880.11	2407.45	0.8457
Cytoplasm	4694.75	1556.58	4240.83	1254.75	0.3750
Tumor mask	6004.00	1598.71	5511.37	1619.80	0.4316
CAIX					
Nuclear	3939.81	1273.12	2406.95	977.10	0.0234
Cytoplasm	3931.01	1699.38	2037.18	944.15	0.0391
Tumor mask	3972.93	1569.58	2124.05	926.91	0.0156
GLUT1					
Nuclear	5389.90	2886.98	2937.34	1198.24	0.0781
Cytoplasm	4932.26	3106.79	2496.81	1116.73	0.1094
Tumor mask	5054.19	2998.35	2658.88	1128.22	0.0469
HIF-1*α*					
Nuclear	6267.08	1603.95	5787.91	2241.58	0.4609
Cytoplasm	3857.75	1221.78	2978.52	1433.30	0.1484
Tumor mask	4582.47	1435.49	4009.57	2108.14	0.3828

SD: standard deviation; *p* values are from the Wilcoxon signed-rank test on log-transformed values.

**Table 2:** Correlation of posttreatment biopsy specimen tissue microarray biomarker expression with disease-free survival in 40 large, deep, high-risk soft tissue sarcoma patients treated with neoadjuvant chemoradiation.

Marker	HR (95% CI)	*p* value
ACIS Ki67		
Percentage	1.01 (0.60–1.68)	0.9813
p53		
Nuclear/cytoplasmic ratio	4.13 (1.29–13.17)	0.0167
ATM		
Nuclear	2.28 (0.34–15.32)	0.3971
Cytoplasm	1.86 (0.32–10.79)	0.4889
Tumor mask	1.33 (0.22–8.11)	0.7594
PARP1		
Nuclear	1.38 (0.18–10.62)	0.7596
Cytoplasm	0.53 (0.12–2.33)	0.3982
Tumor mask	0.55 (0.13–2.42)	0.4317
ERCC1		
Nuclear	0.77 (0.06–9.53)	0.8380
Cytoplasm	0.37 (0.11–1.21)	0.0991
Tumor mask	0.30 (0.09–0.97)	0.0443
XPF		
Nuclear	0.01 (0.00–0.92)	0.0457
Cytoplasm	0.25 (0.03–2.35)	0.2271
Tumor mask	0.16 (0.02–1.62)	0.1207
CAIX		
Nuclear	1.23 (0.48–3.13)	0.6713
Cytoplasm	1.33 (0.60–2.95)	0.4881
Tumor mask	1.32 (0.58–3.03)	0.5084
GLUT1		
Nuclear	1.44 (0.50–4.18)	0.4989
Cytoplasm	1.24 (0.52–2.96)	0.6322
Tumor mask	1.26 (0.50–3.20)	0.6212
HIF-1*α*		
Nuclear	0.59 (0.18–1.89)	0.3712
Cytoplasm	0.37 (0.12–1.16)	0.0885
Tumor mask	0.40 (0.14–1.17)	0.0945

HR: hazard ratio; CI: confidence interval.

**Table 3:** Correlation of posttreatment biopsy specimen tissue microarray biomarker expression with distant disease-free survival in 40 large, deep, high-risk soft tissue sarcoma patients treated with neoadjuvant chemoradiation.

Marker	HR (95% CI)	*p* value
ACIS Ki67		
Percentage	1.01 (0.60–1.69)	0.9691
p53		
Nuclear/cytoplasmic ratio	4.16 (1.31–13.23)	0.0159
ATM		
Nuclear	2.32 (0.35–15.59)	0.3859
Cytoplasm	1.93 (0.34–11.05)	0.4583
Tumor mask	1.37 (0.23–8.33)	0.7294
PARP1		
Nuclear	1.37 (0.18–10.68)	0.7611
Cytoplasm	0.52 (0.12–2.34)	0.3971
Tumor mask	0.55 (0.13–2.43)	0.4313
ERCC1		
Nuclear	0.78 (0.06–9.75)	0.8479
Cytoplasm	10.37 (0.11–1.22)	0.1038
Tumor mask	0.30 (0.09–0.98)	0.0455
XPF		
Nuclear	0.01 (0.00–0.93)	0.0462
Cytoplasm	0.27 (0.03–2.43)	0.2415
Tumor mask	0.17 (0.02–1.69)	0.1304
CAIX		
Nuclear	1.21 (0.47–3.12)	0.6885
Cytoplasm	1.32 (0.59–2.94)	0.5005
Tumor mask	1.31 (0.57–3.02)	0.5214
GLUT1		
Nuclear	1.44 (0.50–4.18)	0.4984
Cytoplasm	1.24 (0.52–2.97)	0.6250
Tumor mask	1.27 (0.50–3.20)	0.6168
HIF-1*α*		
Nuclear	0.59 (0.18–1.90)	0.3717
Cytoplasm	0.37 (0.12–1.17)	0.0913
Tumor mask	0.40 (0.14–1.17)	0.0951

HR: hazard ratio; CI: confidence interval.

## Supplementary Materials

Supplementary 1 Figure A: representative AQUA staining for CAIX (a) and GLUT1 (b) in the soft tissue sarcoma tissue microarray specimens.

Supplementary 2 Table A: the primary fluorescent antibodies and conditions for the heat-induced epitope retrieval (HIER) for the soft tissue sarcoma tissue microarray staining.

Supplementary 3 Table B: clinical and pathologic data for the 51 large, deep, “high-risk” soft tissue sarcoma patients in the TMA study (from RTOG 9514 and the MGH pilot trial).

Supplementary 4 Table C: follow-up and outcome data for the 51 large, deep, “high-risk” soft tissue sarcoma patients in the TMA study (from RTOG 9514 and the MGH pilot trial).

Supplementary 5 Table D: the pretreatment (PRE) group biomarker expression data.

Supplementary 6 Table E: the posttreatment (POST) group biomarker expression data.
