# Supplementary material for: Correlation of High-Risk Soft Tissue Sarcoma Biomarker Expression Patterns with Outcome following Neoadjuvant Chemoradiation
Source: Sarcoma. 2018 Feb 28;2018:8310950. doi: 10.1155/2018/8310950 (PMC5851029; doi:10.1155/2018/8310950)

***Supplemental Material***

**Figure A.** Representative AQUA staining for CAIX (a) and GLUT1 (b) in the soft tissue sarcoma tissue microarray specimens.

a.


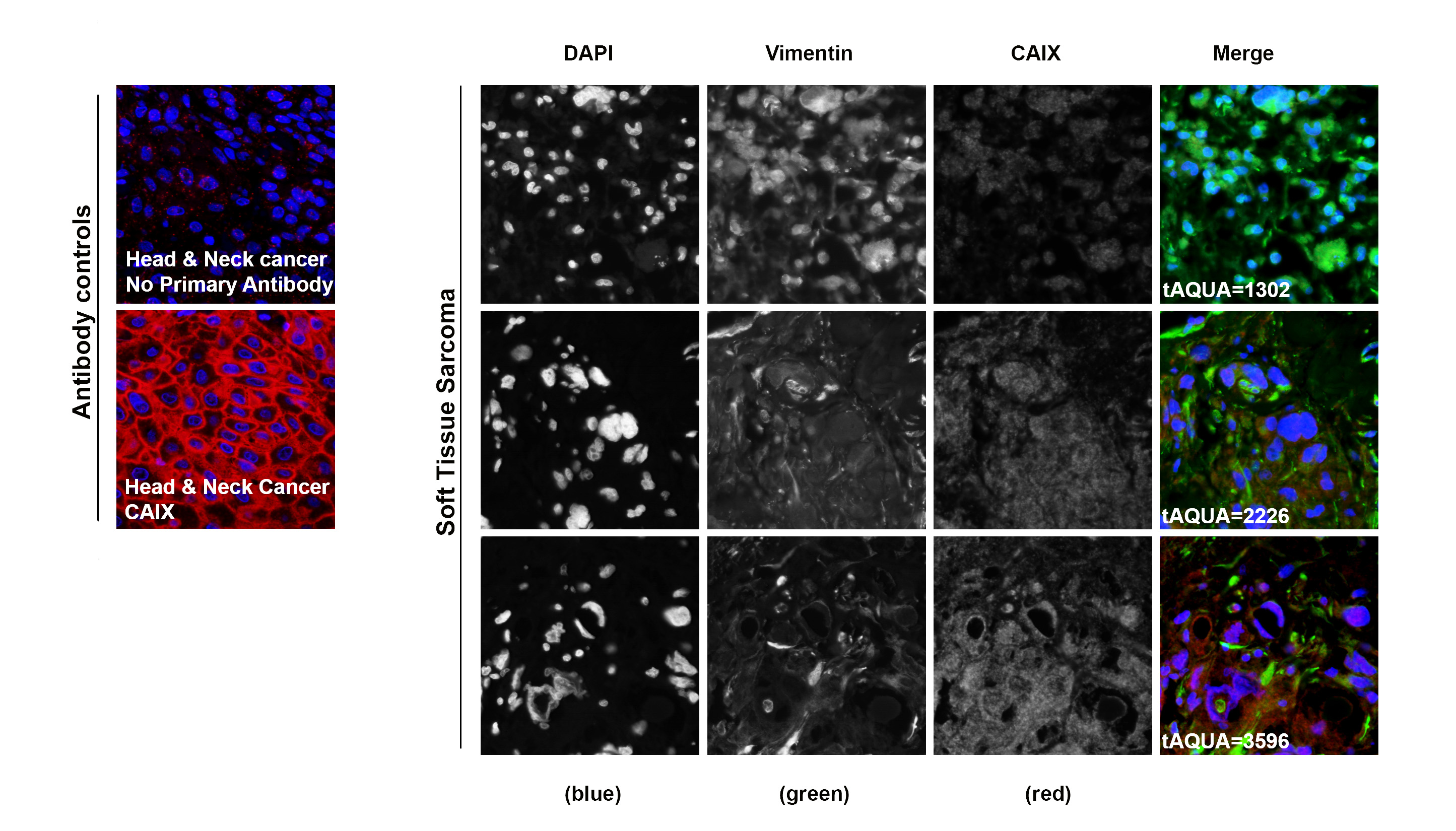


b.


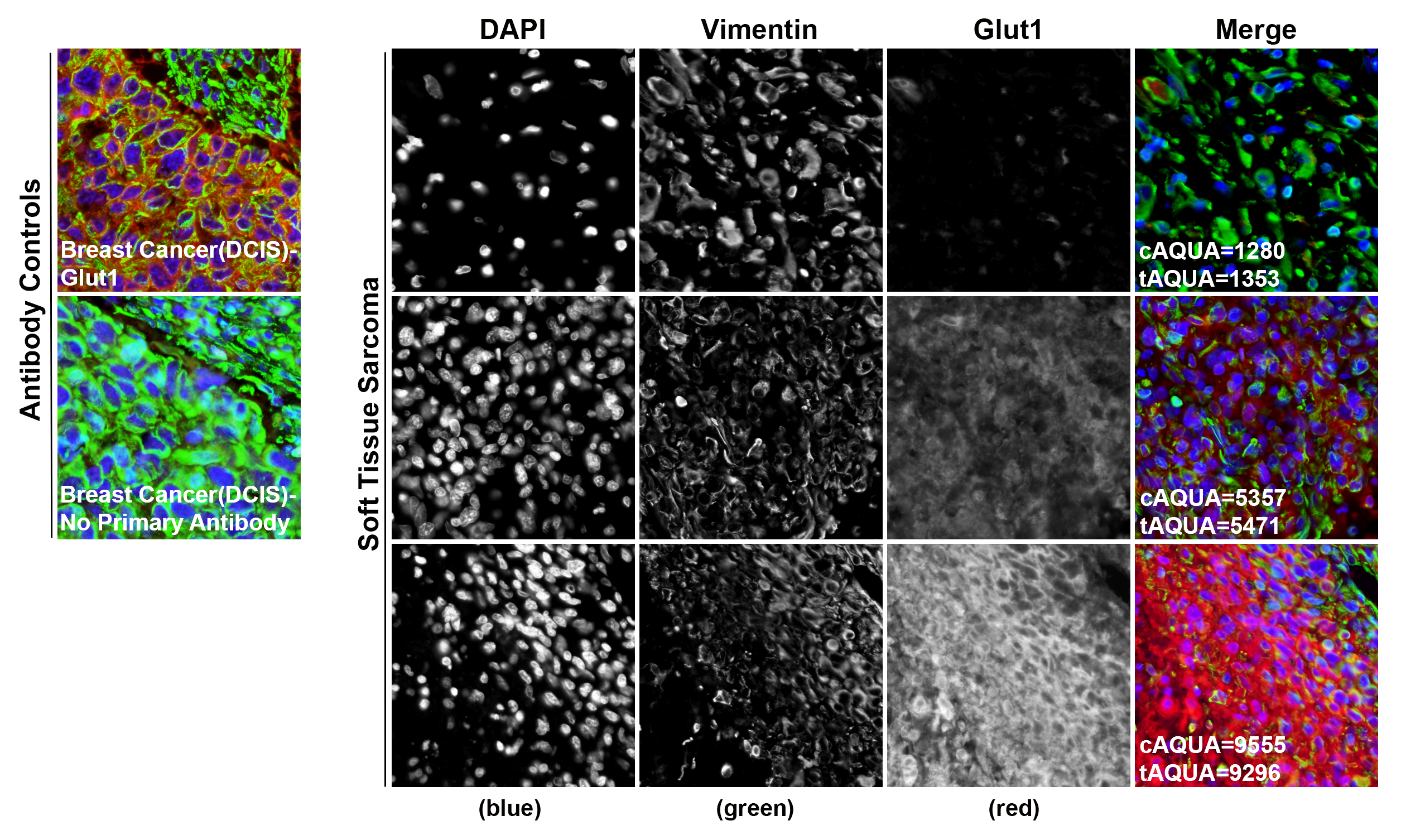

Supplement: Supplementary 1 — Figure A: representative AQUA staining for CAIX (a) and GLUT1 (b) in the soft tissue sarcoma tissue microarray specimens. [file 8310950.f1.docx]
