# Supplementary material for: Correlation of High-Risk Soft Tissue Sarcoma Biomarker Expression Patterns with Outcome following Neoadjuvant Chemoradiation
Source: Sarcoma. 2018 Feb 28;2018:8310950. doi: 10.1155/2018/8310950 (PMC5851029; doi:10.1155/2018/8310950)
Supplement: Supplementary 2 — Table A: the primary fluorescent antibodies and conditions for the heat-induced epitope retrieval (HIER) for the soft tissue sarcoma tissue microarray staining. [file 8310950.f2.docx]

***Supplemental Material***

**Table A.** The primary fluorescent antibodies and conditions for the heat-induced epitope retrieval (HIER) for the soft tissue sarcoma tissue microarray staining.

| **Antibody** | **Manufacturer** | **Product#** | **Host** | **Clone** | **Dilution** | **HIER - Target Retrieval Solution** | **HIER - Time at 121°C** |
| --- | --- | --- | --- | --- | --- | --- | --- |
| ATM | Epitomics | 1549-1 | Rabbit | Y170 | 1:200 | Tris/EDTA | 6 min |
| CAIX | Abcam | ab15086 | Rabbit | Polyclonal | 1:5000 | Citrate | 6 min |
| ERCC1 | Spring Bioscience | M3680 | Rabbit | SP68 | 1:1000 | Citrate | 3 min |
| PARP1 | Epitomics | 1072-1 | Rabbit | E102 | 1:200 | Citrate | 6 min |
| p53 | Epitomics | 1026-1 | Rabbit | E26 | 1:1000 | Citrate | 6 min |
| Hif1a | Epitomics | 2015-1 | Rabbit | EP1215Y | 1:200 | Citrate | 6 min |
| Glut1 | Spring Bioscience | E13810 | Mouse | SPM498 | 1:500 | Tris/EDTA | 3 min |
| XPF | Abcam | ab85140 | Mouse | 3F2/3 | 1:200 | Citrate | 6 min |
|  |  |  |  |  |  |  |  |
| Epitomics: Burlingame, CA; Spring Biosciences: Pleasanton, C; Abcam: Cambridge, MA | | | | | | | |
