# Supplementary material for: Correlation of High-Risk Soft Tissue Sarcoma Biomarker Expression Patterns with Outcome following Neoadjuvant Chemoradiation
Source: Sarcoma. 2018 Feb 28;2018:8310950. doi: 10.1155/2018/8310950 (PMC5851029; doi:10.1155/2018/8310950)
Supplement: Supplementary 3 — Table B: clinical and pathologic data for the 51 large, deep, “high-risk” soft tissue sarcoma patients in the TMA study (from RTOG 9514 and the MGH pilot trial). [file 8310950.f3.docx]

***Supplemental Material***

**Table B**. Clinical and pathologic data for the 51 large, deep, “high risk” soft tissue sarcoma patients in the TMA study (from RTOG 9514 and the MGH pilot trial).

|  | RTOG 9514 (n=29) | MGH (n=22) | Total (n=51) |
| --- | --- | --- | --- |
|  | | | |
| Age (years) |  |  |  |
| Median | 47 | 51.5 | 48 |
| Range | 21 - 75 | 28 - 77 | 21 - 77 |
|  | | | |
| Gender |  |  |  |
| Male | 17 ( 58.6%) | 12 ( 54.5%) | 29 ( 56.9%) |
| Female | 12 ( 41.4%) | 10 ( 45.5%) | 22 ( 43.1%) |
|  | | | |
| Race |  |  |  |
| White | 24 ( 82.8%) | 20 ( 90.9%) | 44 ( 86.3%) |
| African-American | 5 ( 17.2%) | 1 ( 4.5%) | 6 ( 11.8%) |
| Unknown | 0 ( 0.0%) | 1 ( 4.5%) | 1 ( 2.0%) |
|  | | | |
| Histology |  |  |  |
| Epithelioid sarcoma | 1 ( 3.4%) | 0 ( 0.0%) | 1 ( 2.0%) |
| Fibrosarcoma | 4 ( 13.8%) | 1 ( 4.5%) | 5 ( 9.8%) |
| Leiomyosarcoma | 3 ( 10.3%) | 3 ( 13.6%) | 6 ( 11.7%) |
| Liposarcoma (myxoid/round cell, dedifferentiated,pleiomorphic) | 2 ( 6.9%) | 5 ( 22.7%) | 7 ( 13.7%) |
| Undifferentiated pleomorphic sarcoma (MFH) | 12 ( 41.4%) | 11 ( 50.0%) | 23 ( 45.1%) |
| Malignant peripheral nerve sheath tumor | 3 ( 10.3%) | 0 ( 0.0%) | 3 ( 5.9%) |
| Mesenchymal chondrosarcoma | 0 ( 0.0%) | 1 ( 4.5%) | 1 ( 2.0%) |
| Other/unclassified | 3 ( 10.3%) | 0 ( 0.0%) | 3 ( 5.9%) |
| Synovial sarcoma | 1 ( 3.4%) | 1 ( 4.5%) | 2 ( 3.9%) |
|  | | | |
| Histologic grade |  |  |  |
| Grade II | 5 ( 17.2%) | 9 ( 40.9%) | 14 ( 27.5%) |
| Grade III | 24 ( 82.8%) | 13 ( 59.1%) | 37 ( 72.5%) |
|  | | | |
| Tumor location |  |  |  |
| Lower extremity/buttocks | 19 ( 65.5%) | 20 ( 90.9%) | 39 ( 76.5%) |
| Upper extremity | 4 ( 13.8%) | 2 ( 9.1%) | 6 ( 11.8%) |
| Other | 6 ( 20.7%) | 0 ( 0.0%) | 6 ( 11.8%) |
|  | | | |
| Maximal tumor size (longest diameter, cm) |  |  |  |
| Median | 15 | 14 | 14 |
| Range | 8.2 - 24 | 8.5 - 35 | 8.2 - 35 |
|  |  |  |  |
| Radiation dose (Gy), preoperative |  |  |  |
| 28 | 1 ( 3.4%) | 0 ( 0.0%) | 1 ( 2.0%) |
| 41.8-46.2 (44 +/- 5%) | 26 ( 89.7%) | 20 ( 90.9%) | 46 ( 90.2%) |
| 46.79 | 0 ( 0.0%) | 1 ( 4.5%) | 1 ( 2.0%) |
| 48 | 0 ( 0.0%) | 1 ( 4.5%) | 1 ( 2.0%) |
| 50 | 2 ( 6.9%) | 0 ( 0.0%) | 2 ( 3.9%) |
|  |  |  |  |
| Radiation dose (Gy), postoperative |  |  |  |
| 0 | 26 ( 89.7%) | 14 ( 63.6%) | 40 ( 78.4%) |
| 10.8 | 0 ( 0.0%) | 1 ( 4.5%) | 1 ( 2.0%) |
| 15.2-16.8 (16 +/- 5%) | 2 ( 6.9%) | 7 ( 31.8%) | 9 ( 17.6%) |
| 22 | 1 ( 3.4%) | 0 ( 0.0%) | 1 ( 2.0%) |
|  |  |  |  |
| MAID chemotherapy cycles, preoperative |  |  |  |
| 0 | 1 ( 3.4%) | 0 ( 0.0%) | 1 ( 2.0%) |
| 1 | 2 ( 6.9%) | 2 ( 9.1%) | 4 ( 7.8%) |
| 2 | 4 ( 13.8%) | 0 ( 0.0%) | 4 ( 7.8%) |
| 3 | 22 ( 75.9%) | 20 ( 90.9%) | 42 ( 82.4%) |
|  |  |  |  |
| MAID chemotherapy cycles, postoperative |  |  |  |
| 0 | 9 ( 31.0%) | 4 ( 18.2%) | 13 ( 25.5%) |
| 1 | 3 ( 10.3%) | 1 ( 4.5%) | 4 ( 7.8%) |
| 2 | 1 ( 3.4%) | 0 ( 0.0%) | 1 ( 2.0%) |
| 3 | 16 ( 55.2%) | 17 ( 77.3%) | 33 ( 64.7%) |
|  |  |  |  |
| Surgical margin status |  |  |  |
| Positive | 3 ( 10.3%) | 2 ( 9.1%) | 5 ( 9.8%) |
| Negative | 26 ( 89.7%) | 20 ( 90.9%) | 46 ( 90.2%) |
