# Supplementary material for: Correlation of High-Risk Soft Tissue Sarcoma Biomarker Expression Patterns with Outcome following Neoadjuvant Chemoradiation
Source: Sarcoma. 2018 Feb 28;2018:8310950. doi: 10.1155/2018/8310950 (PMC5851029; doi:10.1155/2018/8310950)
Supplement: Supplementary 4 — Table C: follow-up and outcome data for the 51 large, deep, “high-risk” soft tissue sarcoma patients in the TMA study (from RTOG 9514 and the MGH pilot trial). [file 8310950.f4.docx]

***Supplemental Material***

**Table C**. Follow-up and outcome data for the 51 large, deep, “high risk” soft tissue sarcoma patients in the TMA study (from RTOG 9514 and the MGH pilot trial).

|  | RTOG 9514 (n=29) | MGH (n=22) | Total (n=51) |
| --- | --- | --- | --- |
|  | | | |
| Follow-up (years after surgery) |  |  |  |
| Median (range), all patients | 6.65 (0.23-9.02) | 9.70 (0.83-17.58) | 7.27 (0.23-17.58) |
| Median (range), surviving patients | 7.37 (1.75-9.02) | 11.70 (3.39-17.58) | 7.80 (1.75-17.58) |
|  | | | |
| Overall survival |  |  |  |
| Number of events | 8 | 9 | 17 |
| 5-year estimate (95%CI) | 78.8% (63.7-93.9) | 81.1% (64.3-97.8) | 79.9% (68.8-91.1) |
|  | | | |
| Disease-free survival |  |  |  |
| Number of events | 9 | 10 | 19 |
| 5-year estimate (95%CI) | 69.0% (52.1-85.8) | 71.9% (52.7-91.1) | 70.4% (57.9-83.0) |
|  | | | |
| Distant disease-free survival |  |  |  |
| Number of events | 9 | 10 | 19 |
| 5-year estimate (95%CI) | 69.0% (52.1-85.8) | 71.9% (52.7-91.1) | 70.4% (57.9-83.0) |
|  | | | |
| CI: confidence interval. | | | |
