# Supplementary material for: Correlation of High-Risk Soft Tissue Sarcoma Biomarker Expression Patterns with Outcome following Neoadjuvant Chemoradiation
Source: Sarcoma. 2018 Feb 28;2018:8310950. doi: 10.1155/2018/8310950 (PMC5851029; doi:10.1155/2018/8310950)
Supplement: Supplementary 5 — Table D: the pretreatment (PRE) group biomarker expression data. [file 8310950.f5.docx]

***Supplemental Material***

**Table D**. The pre-treatment (PRE) group biomarker expression data.

| Marker | Study | n | Mean | SD | Min | Q1 | Median | Q3 | Max |
| --- | --- | --- | --- | --- | --- | --- | --- | --- | --- |
|  | | | | | | | | | |
| ACIS Ki67 percentage | RTOG 9514 | 16 | 29.72 | 21.97 | 3.29 | 10.07 | 31.90 | 43.95 | 72.28 |
|  | MGH | 5 | 6.58 | 7.24 | 0.67 | 2.35 | 2.55 | 9.19 | 18.15 |
|  | Total | 21 | 24.21 | 21.78 | 0.67 | 8.88 | 16.39 | 36.70 | 72.28 |
|  | | | | | | | | | |
| ATM nuclear AQUA norm | RTOG 9514 | 14 | 8933.81 | 2098.41 | 5995.09 | 7781.62 | 8718.22 | 9992.33 | 12564.24 |
|  | MGH | 4 | 7564.51 | 990.13 | 6612.61 | 6726.79 | 7505.48 | 8402.23 | 8634.48 |
|  | Total | 18 | 8629.52 | 1970.63 | 5995.09 | 6840.97 | 8427.08 | 9893.43 | 12564.24 |
|  | | | | | | | | | |
| ATM cytoplasm AQUA norm | RTOG 9514 | 14 | 4325.63 | 1824.94 | 1976.87 | 3133.97 | 4182.79 | 5175.50 | 8426.90 |
|  | MGH | 4 | 3185.32 | 1473.39 | 2032.83 | 2217.74 | 2696.89 | 4152.90 | 5314.67 |
|  | Total | 18 | 4072.23 | 1779.85 | 1976.87 | 2456.98 | 3728.94 | 5175.50 | 8426.90 |
|  | | | | | | | | | |
| ATM tumor mask AQUA norm | RTOG 9514 | 14 | 5689.13 | 2115.40 | 2848.34 | 4140.16 | 5291.76 | 6814.49 | 10766.80 |
|  | MGH | 4 | 4455.15 | 1429.10 | 3584.05 | 3634.41 | 3825.78 | 5275.88 | 6584.98 |
|  | Total | 18 | 5414.91 | 2015.21 | 2848.34 | 3942.56 | 4922.53 | 6678.10 | 10766.80 |
|  | | | | | | | | | |
| CAIX nuclear AQUA norm | RTOG 9514 | 11 | 3192.56 | 1506.95 | 1282.26 | 2349.04 | 2959.62 | 3896.12 | 6822.00 |
|  | MGH | 4 | 3400.35 | 523.60 | 2981.63 | 3010.66 | 3251.57 | 3790.05 | 4116.66 |
|  | Total | 15 | 3247.97 | 1299.95 | 1282.26 | 2404.59 | 3039.70 | 3896.12 | 6822.00 |
|  | | | | | | | | | |
| CAIX cytoplasm AQUA norm | RTOG 9514 | 11 | 3112.96 | 1874.89 | 1070.37 | 1568.50 | 2761.54 | 3795.00 | 7714.90 |
|  | MGH | 4 | 3338.15 | 521.93 | 2650.64 | 3005.06 | 3391.76 | 3671.24 | 3918.44 |
|  | Total | 15 | 3173.01 | 1606.20 | 1070.37 | 2296.51 | 2762.10 | 3795.00 | 7714.90 |
|  | | | | | | | | | |
| CAIX tumor mask AQUA norm | RTOG 9514 | 11 | 3143.31 | 1790.64 | 1140.62 | 1675.82 | 2877.93 | 3861.28 | 7601.43 |
|  | MGH | 4 | 3417.49 | 418.08 | 2914.18 | 3134.63 | 3411.99 | 3700.35 | 3931.79 |
|  | Total | 15 | 3216.43 | 1530.85 | 1140.62 | 2326.22 | 3084.59 | 3861.28 | 7601.43 |
|  | | | | | | | | | |
| ERCC1 nuclear AQUA norm | RTOG 9514 | 13 | 10502.96 | 2529.16 | 6259.90 | 8931.87 | 10409.96 | 12189.08 | 14668.41 |
|  | MGH | 4 | 7945.55 | 1158.32 | 6919.43 | 7159.82 | 7641.49 | 8731.29 | 9579.81 |
|  | Total | 17 | 9901.22 | 2509.86 | 6259.90 | 7882.77 | 9605.04 | 11345.76 | 14668.41 |
|  | | | | | | | | | |
| ERCC1 cytoplasm AQUA norm | RTOG 9514 | 13 | 3493.28 | 1649.61 | 1574.67 | 2271.03 | 2476.18 | 5117.96 | 5885.38 |
|  | MGH | 4 | 2158.34 | 573.20 | 1360.42 | 1797.37 | 2275.20 | 2519.32 | 2722.55 |
|  | Total | 17 | 3179.18 | 1563.08 | 1360.42 | 2234.32 | 2431.80 | 5093.29 | 5885.38 |
|  | | | | | | | | | |
| ERCC1 tumor mask AQUA norm | RTOG 9514 | 13 | 5683.28 | 1709.54 | 3118.58 | 4339.52 | 5073.64 | 7429.52 | 8015.46 |
|  | MGH | 4 | 3971.43 | 441.65 | 3587.50 | 3676.16 | 3849.66 | 4266.70 | 4598.90 |
|  | Total | 17 | 5280.49 | 1669.94 | 3118.58 | 3934.49 | 4839.21 | 7000.19 | 8015.46 |
|  | | | | | | | | | |
| ERCC1 nuclear/cytoplasm ratio | RTOG 9514 | 13 | 3.51 | 1.50 | 1.88 | 2.47 | 2.98 | 3.92 | 6.61 |
|  | MGH | 4 | 3.94 | 1.38 | 2.72 | 2.91 | 3.62 | 4.97 | 5.79 |
|  | Total | 17 | 3.61 | 1.44 | 1.88 | 2.55 | 3.10 | 4.14 | 6.61 |
|  | | | | | | | | | |
| Glut1 nuclear AQUA norm | RTOG 9514 | 15 | 4626.86 | 2282.38 | 522.33 | 1958.14 | 5334.42 | 6468.55 | 7454.75 |
|  | MGH | 4 | 6687.89 | 2853.62 | 3897.87 | 4283.20 | 6526.55 | 9092.57 | 9800.56 |
|  | Total | 19 | 5060.76 | 2480.74 | 522.33 | 3897.87 | 5334.42 | 7046.84 | 9800.56 |
|  | | | | | | | | | |
| Glut1 cytoplasm AQUA norm | RTOG 9514 | 15 | 5255.34 | 2976.32 | 506.99 | 1869.30 | 5534.59 | 7773.27 | 10077.89 |
|  | MGH | 4 | 5775.51 | 3507.18 | 2504.39 | 2799.04 | 5521.26 | 8751.97 | 9555.11 |
|  | Total | 19 | 5364.85 | 2997.90 | 506.99 | 2504.39 | 5534.59 | 7948.83 | 10077.89 |
|  | | | | | | | | | |
| Glut1 tumor mask AQUA norm | RTOG 9514 | 15 | 5094.09 | 2811.27 | 564.34 | 1882.85 | 5312.74 | 7151.78 | 9674.45 |
|  | MGH | 4 | 6064.86 | 3275.93 | 2810.98 | 3262.14 | 6076.22 | 8867.58 | 9296.02 |
|  | Total | 19 | 5298.46 | 2846.21 | 564.34 | 2810.98 | 5312.74 | 8198.82 | 9674.45 |
|  | | | | | | | | | |
| p53 nuclear AQUA norm | RTOG 9514 | 12 | 1590.10 | 1242.50 | 501.13 | 813.63 | 983.47 | 2217.98 | 3987.19 |
|  | MGH | 4 | 957.60 | 233.57 | 736.55 | 755.78 | 966.82 | 1159.42 | 1160.21 |
|  | Total | 16 | 1431.97 | 1105.91 | 501.13 | 763.80 | 983.47 | 1293.64 | 3987.19 |
|  | | | | | | | | | |
| p53 cytoplasm AQUA norm | RTOG 9514 | 12 | 703.39 | 444.45 | 164.67 | 416.41 | 605.48 | 834.11 | 1784.89 |
|  | MGH | 4 | 676.72 | 166.22 | 507.73 | 567.11 | 647.49 | 786.34 | 904.18 |
|  | Total | 16 | 696.73 | 387.98 | 164.67 | 478.75 | 647.49 | 834.11 | 1784.89 |
|  | | | | | | | | | |
| p53 tumor mask AQUA norm | RTOG 9514 | 12 | 903.49 | 576.22 | 366.47 | 472.89 | 769.56 | 1085.23 | 2207.77 |
|  | MGH | 4 | 759.23 | 168.43 | 581.27 | 619.73 | 755.27 | 898.74 | 945.13 |
|  | Total | 16 | 867.43 | 503.31 | 366.47 | 540.51 | 769.56 | 1009.58 | 2207.77 |
|  | | | | | | | | | |
| p53 nuclear/cytoplasm ratio | RTOG 9514 | 12 | 2.49 | 1.66 | 1.17 | 1.46 | 1.83 | 2.68 | 5.88 |
|  | MGH | 4 | 1.43 | 0.25 | 1.18 | 1.23 | 1.40 | 1.63 | 1.73 |
|  | Total | 16 | 2.22 | 1.50 | 1.17 | 1.40 | 1.59 | 2.10 | 5.88 |
|  | | | | | | | | | |
| PARP1 nuclear AQUA norm | RTOG 9514 | 10 | 6514.22 | 2128.01 | 3578.48 | 4745.99 | 7071.12 | 7854.65 | 9276.09 |
|  | MGH | 4 | 6327.80 | 889.42 | 5119.23 | 5704.78 | 6490.76 | 6950.82 | 7210.46 |
|  | Total | 14 | 6460.96 | 1823.52 | 3578.48 | 5088.45 | 6662.37 | 7747.60 | 9276.09 |
|  | | | | | | | | | |
| PARP1 cytoplasm AQUA norm | RTOG 9514 | 10 | 2344.32 | 1242.61 | 636.27 | 1496.53 | 2022.12 | 3639.06 | 4501.61 |
|  | MGH | 4 | 2887.79 | 563.28 | 2102.77 | 2485.90 | 3040.27 | 3289.68 | 3367.84 |
|  | Total | 14 | 2499.60 | 1098.69 | 636.27 | 1600.22 | 2330.31 | 3367.84 | 4501.61 |
|  | | | | | | | | | |
| PARP1 tumor mask AQUA norm | RTOG 9514 | 10 | 3339.97 | 1528.02 | 1578.27 | 1849.65 | 3486.37 | 4062.77 | 6368.74 |
|  | MGH | 4 | 3924.36 | 600.34 | 3368.49 | 3505.30 | 3786.20 | 4343.42 | 4756.54 |
|  | Total | 14 | 3506.94 | 1332.16 | 1578.27 | 2251.72 | 3752.62 | 4062.77 | 6368.74 |
|  | | | | | | | | | |
| XPF nuclear AQUA norm | RTOG 9514 | 14 | 8130.88 | 1335.37 | 6035.97 | 6917.35 | 8262.89 | 9328.43 | 9942.46 |
|  | MGH | 4 | 8887.49 | 1221.45 | 7574.81 | 8046.28 | 8735.81 | 9728.70 | 10503.53 |
|  | Total | 18 | 8299.02 | 1315.93 | 6035.97 | 7070.68 | 8682.90 | 9328.43 | 10503.53 |
|  | | | | | | | | | |
| XPF cytoplasm AQUA norm | RTOG 9514 | 14 | 4461.08 | 1090.32 | 3206.05 | 3414.91 | 4163.10 | 5402.63 | 6364.70 |
|  | MGH | 4 | 4601.55 | 2201.92 | 2527.43 | 3250.03 | 4082.90 | 5953.08 | 7712.99 |
|  | Total | 18 | 4492.30 | 1329.77 | 2527.43 | 3414.91 | 4153.96 | 5402.63 | 7712.99 |
|  | | | | | | | | | |
| XPF tumor mask AQUA norm | RTOG 9514 | 14 | 5554.28 | 1269.12 | 4062.28 | 4512.95 | 5127.84 | 6907.31 | 7791.48 |
|  | MGH | 4 | 5883.98 | 2226.84 | 3747.40 | 4441.41 | 5395.15 | 7326.56 | 8998.24 |
|  | Total | 18 | 5627.55 | 1458.31 | 3747.40 | 4512.95 | 5213.14 | 6907.31 | 8998.24 |
|  | | | | | | | | | |
| Hif1a nuclear AQUA norm | RTOG 9514 | 13 | 6266.55 | 3481.97 | 1479.64 | 4601.79 | 5748.90 | 6641.15 | 15973.89 |
|  | MGH | 5 | 5474.87 | 3215.89 | 945.96 | 4593.31 | 4818.97 | 7717.83 | 9298.29 |
|  | Total | 18 | 6046.64 | 3335.37 | 945.96 | 4593.31 | 5414.75 | 7717.83 | 15973.89 |
|  | | | | | | | | | |
| Hif1a cytoplasm AQUA norm | RTOG 9514 | 13 | 3477.57 | 2914.61 | 1214.74 | 1794.41 | 2608.94 | 4147.14 | 12358.93 |
|  | MGH | 5 | 3499.51 | 2017.16 | 814.24 | 2952.13 | 3220.29 | 4132.75 | 6378.13 |
|  | Total | 18 | 3483.66 | 2637.03 | 814.24 | 1794.41 | 2959.48 | 4147.14 | 12358.93 |
|  | | | | | | | | | |
| Hif1a tumor mask AQUA norm | RTOG 9514 | 13 | 4284.97 | 3257.46 | 1304.80 | 2630.59 | 3239.32 | 5244.49 | 14087.16 |
|  | MGH | 5 | 4093.96 | 2415.66 | 860.51 | 3103.41 | 4353.20 | 4665.95 | 7486.74 |
|  | Total | 18 | 4231.92 | 2978.41 | 860.51 | 2630.59 | 3368.95 | 5244.49 | 14087.16 |
|  | | | | | | | | | |
| SD: standard deviation; Q1: first quartile; Q3: third quartile. | | | | | | | | | |
