# Supplementary material for: Correlation of High-Risk Soft Tissue Sarcoma Biomarker Expression Patterns with Outcome following Neoadjuvant Chemoradiation
Source: Sarcoma. 2018 Feb 28;2018:8310950. doi: 10.1155/2018/8310950 (PMC5851029; doi:10.1155/2018/8310950)
Supplement: Supplementary 6 — Table E: the posttreatment (POST) group biomarker expression data. [file 8310950.f6.docx]

***Supplemental Material***

**Table E**. The post-treatment (POST) group biomarker expression data.

| Marker | Study | n | Mean | SD | Min | Q1 | Median | Q3 | Max |
| --- | --- | --- | --- | --- | --- | --- | --- | --- | --- |
|  | | | | | | | | | |
| ACIS Ki67 percentage | RTOG 9514 | 18 | 13.68 | 13.20 | 1.65 | 3.05 | 9.11 | 18.84 | 46.72 |
|  | MGH | 21 | 7.33 | 6.42 | 1.04 | 2.80 | 4.74 | 10.12 | 24.73 |
|  | Total | 39 | 10.26 | 10.48 | 1.04 | 2.80 | 7.55 | 13.76 | 46.72 |
|  | | | | | | | | | |
| ATM nuclear AQUA norm | RTOG 9514 | 16 | 8048.18 | 1500.21 | 5619.96 | 7489.09 | 8090.24 | 8784.95 | 11817.09 |
|  | MGH | 17 | 6296.53 | 1493.13 | 3298.21 | 5974.05 | 6176.39 | 7368.83 | 8909.52 |
|  | Total | 33 | 7145.82 | 1720.47 | 3298.21 | 5976.77 | 7411.63 | 8307.76 | 11817.09 |
|  | | | | | | | | | |
| ATM cytoplasm AQUA norm | RTOG 9514 | 16 | 3075.14 | 942.59 | 1468.45 | 2473.36 | 3077.30 | 3539.00 | 5191.70 |
|  | MGH | 17 | 2236.88 | 405.56 | 1678.10 | 1926.26 | 2289.10 | 2457.36 | 3056.09 |
|  | Total | 33 | 2643.31 | 824.44 | 1468.45 | 1940.04 | 2457.36 | 3056.09 | 5191.70 |
|  | | | | | | | | | |
| ATM tumor mask AQUA norm | RTOG 9514 | 16 | 4119.15 | 1087.53 | 2331.60 | 3367.31 | 4171.50 | 4536.07 | 6119.63 |
|  | MGH | 17 | 3168.35 | 637.78 | 1994.80 | 2886.76 | 3184.11 | 3429.83 | 4515.14 |
|  | Total | 33 | 3629.34 | 995.31 | 1994.80 | 3019.97 | 3429.83 | 4178.33 | 6119.63 |
|  | | | | | | | | | |
| CAIX nuclear AQUA norm | RTOG 9514 | 16 | 3495.96 | 2581.79 | 633.49 | 2051.64 | 2416.68 | 4598.21 | 9822.26 |
|  | MGH | 19 | 2985.48 | 960.29 | 1473.35 | 2064.22 | 3123.99 | 3827.92 | 4569.06 |
|  | Total | 35 | 3218.84 | 1869.62 | 633.49 | 2057.59 | 2878.25 | 3830.24 | 9822.26 |
|  | | | | | | | | | |
| CAIX cytoplasm AQUA norm | RTOG 9514 | 16 | 3442.09 | 3071.72 | 509.48 | 1600.51 | 2126.19 | 5169.41 | 11376.54 |
|  | MGH | 19 | 2386.31 | 855.43 | 1159.43 | 1511.96 | 2704.87 | 3092.23 | 3564.89 |
|  | Total | 35 | 2868.95 | 2198.83 | 509.48 | 1594.23 | 2355.65 | 3255.03 | 11376.54 |
|  | | | | | | | | | |
| CAIX tumor mask AQUA norm | RTOG 9514 | 16 | 3579.07 | 3099.35 | 562.96 | 1795.74 | 2219.59 | 4984.47 | 11649.47 |
|  | MGH | 19 | 2564.20 | 906.89 | 1244.84 | 1808.85 | 2743.98 | 3311.90 | 4015.44 |
|  | Total | 35 | 3028.14 | 2221.82 | 562.96 | 1808.85 | 2445.87 | 3532.82 | 11649.47 |
|  | | | | | | | | | |
| ERCC1 nuclear AQUA norm | RTOG 9514 | 14 | 9625.60 | 1081.67 | 7766.56 | 8515.32 | 9978.47 | 10489.15 | 11068.34 |
|  | MGH | 20 | 7765.81 | 1586.49 | 5173.63 | 6627.03 | 7753.71 | 8237.18 | 11130.51 |
|  | Total | 34 | 8531.60 | 1665.30 | 5173.63 | 7357.43 | 8234.71 | 10209.26 | 11130.51 |
|  | | | | | | | | | |
| ERCC1 cytoplasm AQUA norm | RTOG 9514 | 14 | 1949.86 | 973.68 | 674.43 | 1171.41 | 1816.71 | 2527.96 | 3999.87 |
|  | MGH | 20 | 1486.06 | 699.43 | 656.48 | 960.40 | 1334.94 | 1733.18 | 3009.46 |
|  | Total | 34 | 1677.04 | 841.92 | 656.48 | 1019.52 | 1577.37 | 2049.88 | 3999.87 |
|  | | | | | | | | | |
| ERCC1 tumor mask AQUA norm | RTOG 9514 | 14 | 3678.55 | 1232.44 | 1332.46 | 2799.13 | 3594.48 | 4885.83 | 5749.25 |
|  | MGH | 20 | 3085.08 | 1338.07 | 1127.99 | 2000.55 | 2719.91 | 3887.11 | 6289.40 |
|  | Total | 34 | 3329.45 | 1310.39 | 1127.99 | 2326.75 | 3334.80 | 4287.93 | 6289.40 |
|  | | | | | | | | | |
| ERCC1 nuclear/cytoplasm ratio | RTOG 9514 | 14 | 6.31 | 3.42 | 2.13 | 4.38 | 5.13 | 9.09 | 14.45 |
|  | MGH | 20 | 5.97 | 1.98 | 2.62 | 4.43 | 5.98 | 7.02 | 9.93 |
|  | Total | 34 | 6.11 | 2.63 | 2.13 | 4.38 | 5.77 | 7.13 | 14.45 |
|  | | | | | | | | | |
| Glut1 nuclear AQUA norm | RTOG 9514 | 14 | 2913.00 | 1549.72 | 1128.90 | 1728.32 | 2408.19 | 4045.88 | 6777.31 |
|  | MGH | 19 | 3409.72 | 1463.47 | 1503.32 | 2115.98 | 3278.39 | 4459.93 | 6570.64 |
|  | Total | 33 | 3198.99 | 1497.51 | 1128.90 | 2115.98 | 2667.44 | 4110.57 | 6777.31 |
|  | | | | | | | | | |
| Glut1 cytoplasm AQUA norm | RTOG 9514 | 14 | 2750.64 | 1879.00 | 827.28 | 1438.66 | 2297.66 | 3791.42 | 7604.55 |
|  | MGH | 19 | 2906.69 | 1444.83 | 1123.65 | 1633.70 | 2593.60 | 4238.10 | 5679.96 |
|  | Total | 33 | 2840.49 | 1617.00 | 827.28 | 1514.85 | 2539.92 | 3791.42 | 7604.55 |
|  | | | | | | | | | |
| Glut1 tumor mask AQUA norm | RTOG 9514 | 14 | 2814.88 | 1797.09 | 843.95 | 1522.21 | 2309.62 | 3988.67 | 7279.67 |
|  | MGH | 19 | 3092.50 | 1439.78 | 1252.90 | 1847.62 | 2754.95 | 4408.73 | 5690.41 |
|  | Total | 33 | 2974.72 | 1580.33 | 843.95 | 1766.94 | 2600.51 | 3988.67 | 7279.67 |
|  | | | | | | | | | |
| p53 nuclear AQUA norm | RTOG 9514 | 11 | 1211.92 | 613.65 | 401.74 | 516.40 | 1279.70 | 1561.13 | 2212.16 |
|  | MGH | 19 | 1619.23 | 1393.87 | 597.06 | 699.39 | 1064.09 | 1888.96 | 5929.89 |
|  | Total | 30 | 1469.89 | 1172.87 | 401.74 | 699.39 | 1191.09 | 1848.50 | 5929.89 |
|  | | | | | | | | | |
| p53 cytoplasm AQUA norm | RTOG 9514 | 11 | 497.13 | 103.73 | 320.06 | 426.20 | 529.59 | 604.23 | 632.16 |
|  | MGH | 19 | 670.17 | 206.50 | 404.96 | 504.40 | 658.02 | 803.37 | 1099.83 |
|  | Total | 30 | 606.72 | 193.31 | 320.06 | 463.89 | 548.18 | 713.25 | 1099.83 |
|  | | | | | | | | | |
| p53 tumor mask AQUA norm | RTOG 9514 | 11 | 617.76 | 181.99 | 339.10 | 430.00 | 636.79 | 711.60 | 913.02 |
|  | MGH | 19 | 904.90 | 462.55 | 450.96 | 559.18 | 837.42 | 1127.59 | 2326.98 |
|  | Total | 30 | 799.62 | 405.00 | 339.10 | 556.41 | 666.52 | 925.46 | 2326.98 |
|  | | | | | | | | | |
| p53 nuclear/cytoplasm ratio | RTOG 9514 | 11 | 2.31 | 0.85 | 1.10 | 1.41 | 2.37 | 3.27 | 3.50 |
|  | MGH | 19 | 2.25 | 1.46 | 1.15 | 1.38 | 1.75 | 2.59 | 6.63 |
|  | Total | 30 | 2.27 | 1.25 | 1.10 | 1.41 | 1.82 | 2.59 | 6.63 |
|  | | | | | | | | | |
| PARP1 nuclear AQUA norm | RTOG 9514 | 13 | 6095.11 | 1159.64 | 3902.24 | 5307.36 | 5830.02 | 6874.64 | 7934.26 |
|  | MGH | 18 | 5541.87 | 1639.02 | 3254.74 | 4394.37 | 5363.10 | 6095.65 | 9071.94 |
|  | Total | 31 | 5773.87 | 1461.92 | 3254.74 | 4813.06 | 5492.38 | 6874.64 | 9071.94 |
|  | | | | | | | | | |
| PARP1 cytoplasm AQUA norm | RTOG 9514 | 13 | 2137.70 | 1281.98 | 975.59 | 1347.95 | 1931.02 | 2368.85 | 6066.18 |
|  | MGH | 18 | 2371.15 | 960.84 | 1212.75 | 1724.11 | 2167.35 | 2726.32 | 4543.95 |
|  | Total | 31 | 2273.26 | 1092.82 | 975.59 | 1594.98 | 2029.15 | 2523.25 | 6066.18 |
|  | | | | | | | | | |
| PARP1 tumor mask AQUA norm | RTOG 9514 | 13 | 2987.10 | 1282.02 | 1298.28 | 2192.43 | 2853.15 | 3547.40 | 6406.72 |
|  | MGH | 18 | 3229.48 | 1210.29 | 1534.40 | 2190.25 | 2938.79 | 3936.20 | 5608.66 |
|  | Total | 31 | 3127.84 | 1225.67 | 1298.28 | 2190.25 | 2853.15 | 3766.12 | 6406.72 |
|  | | | | | | | | | |
| XPF nuclear AQUA norm | RTOG 9514 | 17 | 8016.57 | 1992.93 | 6095.47 | 7250.20 | 7567.05 | 8018.98 | 15215.35 |
|  | MGH | 19 | 8455.28 | 1371.33 | 6569.48 | 7322.19 | 8260.02 | 9324.91 | 11625.12 |
|  | Total | 36 | 8248.11 | 1682.90 | 6095.47 | 7286.19 | 7778.64 | 8891.30 | 15215.35 |
|  | | | | | | | | | |
| XPF cytoplasm AQUA norm | RTOG 9514 | 17 | 3818.47 | 1178.17 | 2233.00 | 3334.60 | 3771.62 | 4338.96 | 7225.68 |
|  | MGH | 19 | 3598.13 | 720.17 | 2624.27 | 2914.04 | 3353.94 | 3998.37 | 5245.54 |
|  | Total | 36 | 3702.18 | 955.89 | 2233.00 | 3047.95 | 3583.84 | 4221.85 | 7225.68 |
|  | | | | | | | | | |
| XPF tumor mask AQUA norm | RTOG 9514 | 17 | 4756.24 | 1483.84 | 2717.92 | 4057.78 | 4733.15 | 5223.34 | 9430.09 |
|  | MGH | 19 | 4824.24 | 912.59 | 3588.97 | 4111.84 | 4587.36 | 5329.66 | 6806.78 |
|  | Total | 36 | 4792.13 | 1198.34 | 2717.92 | 4084.81 | 4652.70 | 5242.49 | 9430.09 |
|  | | | | | | | | | |
| Hif1a nuclear AQUA norm | RTOG 9514 | 13 | 4362.21 | 1583.99 | 1618.79 | 3248.35 | 4248.39 | 4966.20 | 7602.49 |
|  | MGH | 19 | 6601.11 | 2439.43 | 2274.81 | 4810.64 | 6498.57 | 8335.45 | 12844.03 |
|  | Total | 32 | 5691.56 | 2382.16 | 1618.79 | 3842.67 | 5502.91 | 7332.17 | 12844.03 |
|  | | | | | | | | | |
| Hif1a cytoplasm AQUA norm | RTOG 9514 | 13 | 1893.30 | 779.93 | 966.06 | 1414.18 | 1668.49 | 2292.86 | 3969.33 |
|  | MGH | 19 | 3403.24 | 1395.52 | 1269.41 | 2394.39 | 3051.16 | 4094.26 | 6565.47 |
|  | Total | 32 | 2789.83 | 1390.66 | 966.06 | 1702.60 | 2438.41 | 3784.14 | 6565.47 |
|  | | | | | | | | | |
| Hif1a tumor mask AQUA norm | RTOG 9514 | 13 | 2368.68 | 932.79 | 1098.09 | 1841.48 | 2355.22 | 2895.31 | 4754.04 |
|  | MGH | 19 | 4421.83 | 1754.38 | 1552.10 | 3119.17 | 4519.56 | 5358.44 | 7781.92 |
|  | Total | 32 | 3587.74 | 1781.45 | 1098.09 | 2190.53 | 3075.78 | 4688.94 | 7781.92 |
|  | | | | | | | | | |
| SD: standard deviation; Q1: first quartile; Q3: third quartile. | | | | | | | | | |
